# Complete genome sequencing of a *Tequintavirus* bacteriophage with a broad host range against *Salmonella Abortus equi* isolates from donkeys

**DOI:** 10.3389/fmicb.2022.938616

**Published:** 2022-08-16

**Authors:** Wenhua Liu, Letian Han, Peng Song, Huzhi Sun, Can Zhang, Ling Zou, Jiaqi Cui, Qiang Pan, Huiying Ren

**Affiliations:** ^1^College of Veterinary Medicine, Qingdao Agricultural University, Qingdao, Shandong, China; ^2^Qingdao Phagepharm Bio-tech Co., Ltd., Qingdao, Shandong, China

**Keywords:** *S. Abortus equi*, *E. coli*, bactericidal activity, complete genome, function of ORFs, abortive rate, phage vB_SabS_Sds2 (Sds2)

## Abstract

Salmonella enterica subspecies enterica serovar abortus equi (*S. Abortus equi*) is the most common cause of abortion in mares. It has recently been found to cause abortion in donkeys more frequently in China. A novel virulent bacteriophage vB_SabS_Sds2 (hereafter designated as Sds2) was isolated from the feces of donkeys using a *S. Abortus equi* strain as a host. Phage Sds2 had an isometric polyhedral head and an uncontracted long tail, belonging to the *Tequintavirus*, *Markadamsvirinae*, *Demerecviridae*, *Caudovirales*. The genome of phage Sds2 was 114,770 bp, with a GC content of 40.26%. The genome contained 160 open reading frames (ORFs), and no ORFs were associated with pathogenicity, drug resistance, or lysogenization by sequence analysis. Both genome annotation and phylogenetic analysis indicated that phage Sds2 was highly similar to T5-like bacteriophages. Phage Sds2 could lyse 100% (30/30) of *S. Abortus equi* strains, 25.3% (24/95) of other serotypes of *Salmonella* strains, and 27.6% (8/29) of *Escherichia coli* strains using the double-layer agar plate method. The *in vitro* test showed that phage Sds2 had high bactericidal activity against *S. Abortus equi* at a wide range of MOIs. The *in vivo* test indicated that phage Sds2 had an inhibitory effect on abortion in mice challenged with *S. Abortus equi.* In general, phage Sds2 is a novel lytic phage with a wide host range and has the potential to prevent abortion caused by *S. Abortus equi.*

## Introduction

*Salmonella* is a genus of the family Enterobacteriaceae that is important for public health and economic development worldwide. Different serotypes of *Salmonella* often have different host specificity (Uzzau et al., 2000). *Salmonella enterica subspecies enterica serovar abortus equi* (*S. Abortus equi*) is a serotype restricted to equines, which is regarded as the most common cause of abortion in mares, orchitis in stallions, and septicemia or polyarthritis in foals (Bustos et al., 2016). Moreover, infected mares could be carriers and disseminators of pathogens, leading to widespread infection (Niwa et al., 2016). *S. Abortus equi* is usually found on farms due to a lack of vaccination and poor husbandry practices (Abhishek et al., 2018). In recent years, an increasing number of abortion cases in mares caused by *S. Abortus equi* have been reported in many countries (Bustos et al., 2016; Stritof et al., 2016; Grandolfo et al., 2018). In China, donkey husbandry is booming due to the increasing demands. However, the high abortion rate caused by *S. Abortus equi* has seriously hampered the development of donkey husbandry (Wang et al., 2019; Zhu et al., 2021).

Antibiotics have been widely used for the prevention and control of bacterial infections. However, antibiotic-resistant bacterial strains are increasingly emerging, and there is an urgent need for effective drugs. In Italy, 16 isolates of *Salmonella* in foals exhibited the same antimicrobial resistance pattern and were resistant to doxycycline, colistin, ciprofloxacin, streptomycin, and sulfamethoxazole (Grandolfo et al., 2018). Furthermore, antibiotic-resistant genes can be transferred from animals to zoonotic pathogens, thereby threatening human health (Tollefson and Flynn, 2002).

Bacteriophages are highly specific viruses that can infect bacteria (Hampton et al., 2020), and they have been considered potential and effective biological control agents. Due to the increasing infections caused by antibiotic-resistant bacteria in animal husbandry, the use of virulent phages as a therapeutic alternative to antibiotics against bacterial infection draws more and more attention (Wang C. et al., 2017). Phage therapy has been used to control *Salmonella* infection (salmonellosis) in various animals, such as swine, and chicken, among others (Seo et al., 2018; Clavijo et al., 2019). Nevertheless, little is known about the use of phage therapy for controlling salmonellosis in donkeys.

In this study, in order to test whether a novel lytic phage contained virulence genes, lysogenic genes, or resistance genes, we analyzed its whole genome sequence. In general, phages are highly host-specific. However, if a phage has a broad host range, it will have great potential for clinical application. To evaluate the applicability of the phage, its host range was tested against both *Salmonella* and *Escherichia coli* strains. Bactericidal activity *in vitro* of the isolated phage against *S. Abortus equi* was tested at different MOIs to find the best ratio. Furthermore, to observe the preventive and protective effects of the phage on abortion caused by *S. Abortus equi* in mice, experiments were designed to detect the bactericidal activity *in vivo*.

## Materials and methods

### Animals

SPF KM mice at 13 gestational day (GD) were purchased from Qingdao Daren Fucheng Animal Husbandry Co., Ltd., Shandong, China. Animal experiments performed in this study strictly followed the national guidelines for experimental animal welfare announced by Ministry of Science and Technology of People’s Republic of China in 2006 (Guiding Opinions on Kindly Treating Laboratory Animals^1^) and were approved by the Animal Welfare and Research Ethics Committee at Qingdao Agricultural University, Shandong, China.

### Bacterial strains and growth condition

Forty-nine *Salmonella* strains of donkey origin used in this study were previously isolated and kept in the Veterinary Microbiology Lab of Qingdao Agricultural University. Among them, 30 strains of *S. Abortus equi* were got from organs such as the liver and the spleen, as well as the placenta of different aborted fetuses in donkey farms located in different regions (Donge, Yucheng, and Xinjiang, among others) of China over the past 4 years, and 19 strains of other serotypes of *Salmonella* were from donkey feces collected from the above-mentioned donkey farms. All the 49 strains were previously identified by Chromogenic *Salmonella* Agar (CHROMagar, France), polymerase chain reaction (PCR), and serotypes (unpublished). Seventy-six strains of other *Salmonella* and 29 strains of *E. coli* were kindly provided by Qingdao Phagepharm Bio-tech Co., Ltd., Qingdao, China. All strains were cultivated in Luria-Bertani (LB) broth at 37?, and stock cultures were stored in the LB broth supplemented with 30% glycerol at -80°C.

### Isolation and purification of phages

Isolation and purification of phages were carried out by the double-layer plate method (Wang S. et al., 2017). Feces were collected from a donkey farm with more than 1,000 female donkeys in Donge, Shandong province of China, where abortion had occurred before. Each feces of about 10 g was mixed with the indicator bacterium of 200 μL *S. Abortus equi* S2 strain (10^9^ CFU/ml) in 100 ml of LB broth and incubated at 37? for 16 h, followed by centrifugation at 11,000 *g* for 10 min. The supernatant was collected and filtered through a 0.22-μm membrane to obtain a phage stock solution. The stock solution of 100 μL was mixed with the same volume of *S. Abortus equi* S2 strain and incubated at 37°C for 5 min. Finally, each mixture (200 μL) was mixed with molten agar (5 ml) at 50? and poured on the top of the LB plates. The plates were incubated at 37°C for 12 h to produce plaques. Individual phage plagues were selected and dissolved in 100 μL of LB broth at 40°C for 30 min, followed by centrifugation at 11,000 *g* for 5 min. The supernatant was diluted and purified using the double-layer agar plate method at least three times to obtain uniform phage plaques.

### Morphological characterization

The morphology of phage Sds2 was examined using transmission electron microscopy (TEM) (Ackermann, 2009). Briefly, 20 μL of the phage suspension (≥10^8^ PFU/ml) was dropped on the surface of carbon-coated copper grids and adsorbed for 15 min. Then, the phages were stained with 2% uranyl acetate in darkness for 10 min and were examined using an HT7700 Transmission Electron Microscope (TEM, Hitachi, Japan) at an accelerating voltage of 80 kV.

### Host range and efficiency of plating investigation

The host range of phage Sds2 was tested using 30 strains of *S. Abortus equi* and 95 strains of other serotypes of *Salmonella* and 29 *E. coli* strains using the double-layer agar plate method.

The tested bacterial strains were freshly prepared at 10^8^ CFU/ml. Phage Sds2 suspensions (100 μL, 10^5^ PFU/ml) were mixed with the same volume of tested bacteria, followed by incubation at 37°C for 5 min. The phage titers were then determined as described above. The efficiency of plating (EOP) values was determined by the ratio of PFUs of phage Sds2 from each susceptible strain to CFUs from the indicator strain of *S. Abortus equi* S2. Each experiment was repeated three times.

### One-step growth curve of phage Sds2

A one-step growth curve was generated as described previously, with some modifications (Lee et al., 2021). Briefly, phage Sds2 was mixed with the indicator strain of *S. Abortus equi* S2 (1.5 × 10^8^ CFU/ml) at a multiplicity of infection (MOI) of 0.1, followed by incubation at 37°C for 5 min. Then, the mixture was centrifuged at 11,000 *g* for 30 s and washed two times with the LB broth to remove unabsorbed phages. The pellet was resuspended in 5 ml of the LB broth, and the cultures were grown at 37°C with shaking at 180 r/min. Aliquots of the cultures (200 μL) were sampled at intervals of 5 min in 1 h, 20 min in 2 h, and 30 min in 3 h after the inoculation of phages. The titer of the phage was detected after centrifugation (11,000 *g*, 5 min). Each aliquot was performed three times. The burst size was calculated as the ratio of the final count of liberated phage particles to the initial count of phage particles.

### Bacteriophage genome extraction

The genomic DNA of phage Sds2 was extracted using a phenol-chloroform method (Zhao F. et al., 2019). Briefly, 600 μL of phage suspension was treated with DNase I and RNase A (1 μg/ml) at 37? for 30 min. The mixture was incubated at 80°C for 15 min to deactivate DNase I. Then, the mixture was incubated with proteinase K (50 μg/ml), SDS (0.5%), and EDTA (20 mM) at 56? for 1 h. The DNA was extracted by adding an equal volume of phenol–chloroform–isoamyl alcohol (25:24:1). After centrifugation at 8000 *g* for 10 min, the aqueous layer was collected, mixed with an equal volume of isopropanol, and maintained at –20°C for 4 h. After centrifugation (11,000 *g*, 20 min), DNA pellets were washed three times with cold 75% ethanol and air-dried at room temperature. Finally, DNA pellets were resuspended in 30 μL of sterile deionized water, and then DNA was verified using the Nanodrop (Agilent 5400, American). The sequencing was conducted by Genomics Solution Limited, SZHT (Shenzhen, China).

### Genome annotation and bioinformatics analysis

The purified genomic DNA was sheared into c. 350 bp fragments to construct a paired-end (PE) library using the Nextera XT sample preparation kit (Illumina, San Diego, CA, United States). The PE reads of 150 bp were generated by a Novaseq 6000 sequencer (Illumina, San Diego, CA, United States). High-quality reads were assembled into the phage genome using the *de novo* assembler SPAdes v.3.11.0 software (Bankevich et al., 2012). The complete sequence was annotated using RAST,^2^ BLASTp, and GeneMark^3^ (Besemer et al., 2001; Aziz et al., 2008). The predicted ORFs were verified using the online BLASTp.^4^ Putative transfer RNA (tRNA) encoding genes were searched using tRNAscan-SE^5^ (Schattner et al., 2005). The phylogenetic tree of phage Sds2 was constructed based on terminase large subunits and major capsid protein using MEGA 6 (Tamura et al., 2013).

### *In vitro* bactericidal activity

In order to know the bactericidal activity of phage Sds2 against *S. Abortus equi*, *S. Abortus equi* S2 was used as the indicator bacteria. First, the phage suspension was serially diluted (10-fold) with the LB broth. Then, 200 μL of each phage dilution was mixed with the same volume of bacterial suspensions (10^8^ CFU/ml) at different MOIs (1, 0.1, 0.01, 0.001, and 0.0001), followed by incubation in 7 ml of LB broth at 37? for 24 h. The optical density at 600 nm (OD_600_) was measured in a 96-well plate using a UV-vis spectrophotometer at intervals of 1 h for the first 10 h, and 24 h. At the same time, a 100-μL culture of 2 h interval was sampled for counting the number of colonies. The bacterial culture without phages served as a positive control, and the LB broth served as a negative control. The experiment was performed three times. All data were statistically analyzed using GraphPad Prism 6.0 software.

### Effect of phage Sds2 on abortion rates in a murine model

*Salmonella enterica subspecies enterica serovar abortus equi* can induce abortion in mice (Wang et al., 2020; Zhu et al., 2021). In this study, SPF pregnant KM mice were used to establish a murine abortion model for detecting the effect on abortion rates of phage Sds2 against *S. Abortus equi* S2 (one host strain of phage Sds2) challenge. Seventy mice at 13 gestational day (GD) were pre-bred for 2 days before the experiment. All mice were randomly divided into 7 groups (groups A to F, 10 mice per group) at 15 GD. Mice in group E were challenged with *S. Abortus equi* S2 intraperitoneally with the dose of 2.6 × 10^5^CFU/mouse, which was the half dose of abortion tested before (data was not published), groups A, B, C, and D were given the same challenge doses as that of group E. Mice in groups A and B (phage preventive group) were treated with phage Sds2 (0.2 ml) intraperitoneally at 3.2 × 10^8^ PFU/mouse and 3.2 × 10^6^ PFU/mouse, respectively, at 1 h before the bacterial challenge. Mice in groups C and D (phage therapeutic group) were injected with phage Sds2 (0.2 ml) intraperitoneally at 3.2 × 10^8^ PFU/mouse and 3.2 × 10^6^ PFU/mouse, respectively, at 1 h after the bacterial challenge. Mice in group F were treated with phage Sds2 (0.2 ml) at 5.6 × 10^8^ PFU/mouse intraperitoneally to detect the safety of phage Sds2 on mice. Mice in group G acted as blank control and were given a treatment of LB broth (0.2 ml) intraperitoneally. Both bacteria and phage were diluted with sterile PBS. All mice were fed under the same condition and monitored at intervals of 2 h daily. When abortive symptoms of vaginal bleeding or giving birth to dead fetus were observed, the abortive mice were humanely euthanized by intravenous injection of pentobarbital sodium (100 mg/kg of body weight) as previously described (Wang et al., 2020). The abortive rates of all groups were calculated when all mice gave birth.

## Results

### Morphology of phage Sds2

The clear plaques produced by the phage from donkey feces that infected *S. Abortus equi* S2 strain had well-defined boundaries with a diameter of about 2 mm (Supplementary Figure 1), and the phage titer against *S. Abortus equi* S2 strain was 2.8 × 10^9^ PFU/ml. TEM images showed that the phage possessed an isometric polyhedral head of about 80 nm in diameter and an uncontracted long tail of approximately 170 nm (Supplementary Figure 2), belonging to *Caudovirales*, and designated as vB_SabS_Sds2 (abbreviated of Sds2).

### Host range and efficiency of plating investigation

Efficiency of plating was calculated to determine the host range of phage Sds2 against the 154 tested bacteria. In terms of lytic capacity, EOP was divided into four levels, namely, high production (EOP ≥ 0.5), medium production (0.1 ≤ EOP < 0.5), low production (0.001 < EOP < 0.1), and no production (EOP ≤ 0.001) (Khan and Nilsson, 2015). According to the above criteria, we found that phage Sds2 exhibited a high lytic activity of 73.3% (22/30) and a medium lytic activity of 26.7% (8/30) to *S. Abortus equi*. In addition, it had a high lytic activity of 15.8% (15/95), a medium lytic activity of 3.2% (3/95), and a low lytic activity of 6.3% (6/95) to other strains of *S. enterica*. Furthermore, phage Sds2 displayed a high lytic activity of 10.3% (3/29), a medium lytic activity of 13.8% (4/29), and a low lytic activity of 3.4% (1/29) to *E. coli* strains (Supplementary Table 1). The results indicated that phage Sds2 could potentially control *Salmonella* and *E. coli* infections.

### One-step growth curve of phage Sds2

**FIGURE 1:**
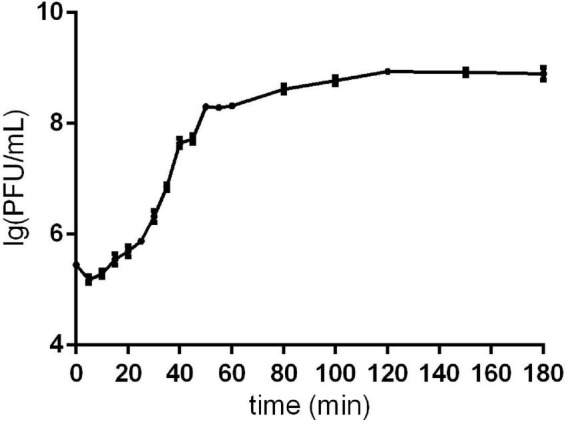
One-step growth curve of phage Sds2. One-step growth curve of phage Sds2. The data are expressed as means ± SD (*n* = 3).

The one-step growth curve showed that phage Sds2 had a latent period of 10 min and a burst period of 30 min. The burst size was approximately 86 PFU/cell (Figure 1).

### Genomic characteristics of phage Sds2

**FIGURE 2:**
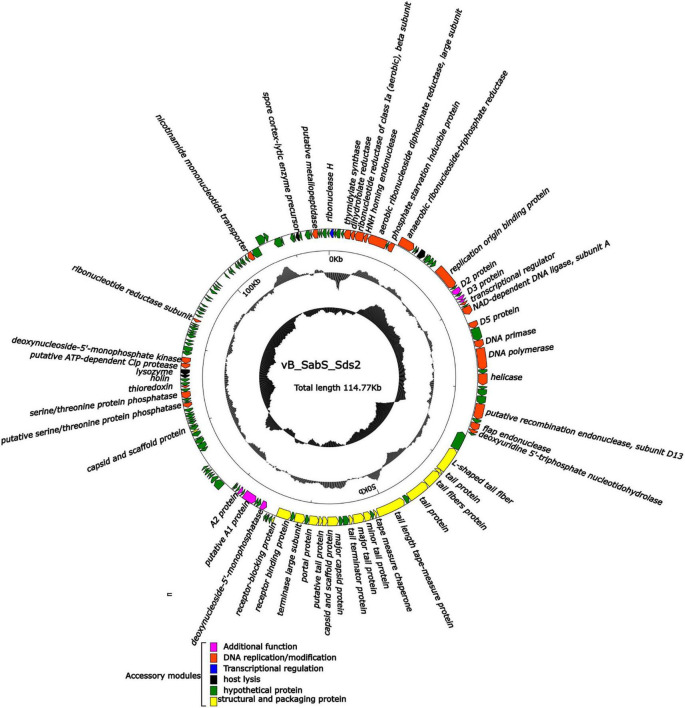
Complete genome structure of phage vB_SabS_Sds2. The outermost circle is the 160 ORFs coded by phage vB-SabS-Sds2. The arrows represent the direction of gene transcription, and the colors represent genes with different functions: structural and packaging protein (yellow); host lysis-related genes (black), DNA replication/modification genes (red); transcriptional regulation genes (blue); hypothetical protein genes (green); and additional function genes (pink).

According to the whole genome sequence analysis, the genome of phage Sds2 was a double-stranded DNA (ds-DNA) molecule and constituted 114, 770 bp. The overall G + C content of phage Sds2 was 40.3%, lower than that of *Salmonella* strains (about 50–52%) (Moreno et al., 2013), while similar to that of T5-like coliphage EPS7 (39%) and T5-like coliphage SPC35 (39.4%). The genome of phage Sds2 contained 160 ORFs, of which 56 ORFs had known protein functions, and the others were annotated to be hypothetical proteins (Supplementary Table 2). In addition, 22 tRNA genes were found in the genome of phage Sds2 (Supplementary Table 3). There were no known lysogeny-related genes, resistance genes, or pathogen-associated genes in phage Sds2. Phage Sds2 possessed almost the same modular genomic structure as ds-DNA phages, especially as T5-like phages (Figure 2). The complete genome sequence of phage Sds2 had been deposited into GenBank (accession number: MW357609).

#### Phylogenetic analysis

**FIGURE 3:**
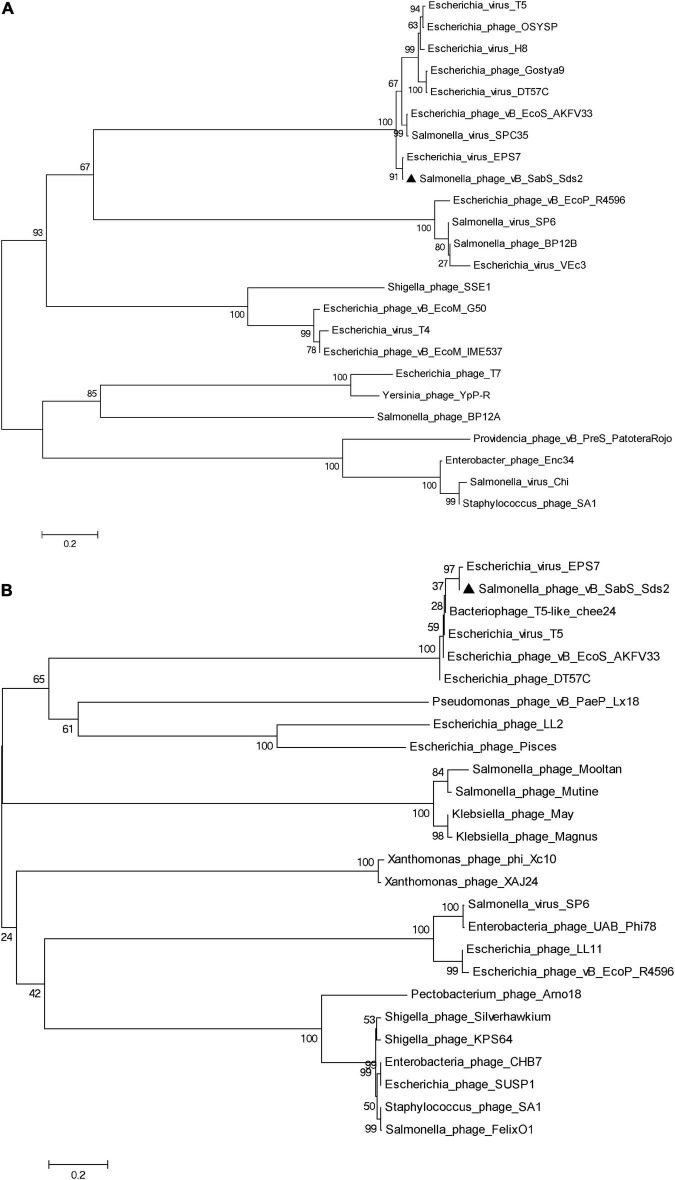
Phylogenetic analysis of phage vB_SabS_Sds2. Phylogenetic tree based on the major capsid protein **(A)**, the terminase large subunit **(B)** were compared using MEGA6, and the phylogenetic tree was generated using the neighbor-joining method and 1,000 bootstrap replicates.

Compared to the genome in the NCBI databases, phage Sds2 was highly homologous to *Salmonella* phage vB_SenS_SB6 (98.4%) and *Salmonella* phage S126 (98.1%). A phylogenetic tree based on terminase large subunits and major capsid protein revealed that phage Sds2 was in the same cluster with T5 and T5-like bacteriophages and was closely related to coliphage EPS7 on the same branch (Figures 3A,B). Based on the sequence, phylogenetic relationship, genomic size, and architecture, phage Sds2 could be classified as a T5-like virus. According to the ICTV virus classification system, phage Sds2 was a member of *Tequintavirus*, *Markadamsvirinae*, *Demerecviridae*, *Caudovirales* (Turner et al., 2021).

#### Functions of open reading frames

The functional genes of phage Sds2 encoded various proteins that were associated with DNA replication and modification, transcriptional regulation, phage packaging and structural proteins, and proteins involved in host lysis. ORF71, ORF74, and ORF76 were involved in the infection of the host immediately after an injection of phage nucleic acid by shutting off the synthesis of host genes and proteins, as well as the degradation of host DNA (Hong et al., 2008; Nilsson, 2014; Davison, 2015). ORF6, ORF11, ORF30, ORF 32, ORF42, and ORF106 encoded enzymes were related to the synthesis of RNA and DNA precursors, promoting the growth of a phage under phosphate starvation conditions, DNA recombination, DNA repair and transcription, initiating the synthesis of DNA, and achieving a high rate of DNA synthesis in hosts (Kim et al., 1993; Wang et al., 2005; Lundin et al., 2009; Petzold et al., 2015). Details are listed in Supplementary Table 2.

The structural proteins, including major capsid protein, capsid and scaffold protein, tail fiber protein, portal protein, terminase large subunits, and scaffold protein, were encoded by many late genes (Supplementary Table 2). Phage Sds2 is equipped with L-shaped tail fibers (ORF45), which is associated with the specifical binding to bacterial lipopolysaccharide (LPS) O-antigen, leading to an increase in the rate of adsorption of phages on O-antigen-producing strains. The receptor recognition by the L-shaped tail fibers is thought to be quite rapid but less specific than the ultimate infection-triggering interaction of central fibers with the corresponding receptors (Golomidova et al., 2016). In natural ecosystems, since the number of hosts is lower than that of phages, the high rate of adsorption can facilitate phages to infect the hosts. ORF65 encoded receptor-binding proteins, which exhibited 97.85, 98.31, and 83.03% nucleotide sequence identity with phage EPS7, phage BSP22A, and phage SPC35, respectively. Since these three phages were BtuB-targeting phages, phage Sds2 might also target BtuB as a receptor (Hong et al., 2008; Kim and Ryu, 2011; Bai et al., 2019). Among 160 ORFs of phage Sds2, 31 ORFs had more than 98.59% homology with coliphage EPS7. Furthermore, three ORFs (ORF46, ORF109, and ORF110) had 100% identities with phage EPS7, which might explain why phage Sds2 could infect both *Salmonella spp.* and *E. coli*.

The genome of phage Sds2 contained 22 tRNA genes (Supplementary Table 3). The distribution of tRNA was highly associated with codon usage. It is reported that tRNA genes benefit the replication of phages by corresponding to codons used by the phage rather than the host (Bailly-Bechet et al., 2007). The extra tRNA genes can minimize host dependence and extend the host spectrum, thereby improving their fitness to different hosts (Rak et al., 2018). Therefore, due to the presence of a large number of tRNAs genes in the genome, phage Sds2 could have a wide host spectrum, which is identified in Supplementary Table 1.

### *In vitro* bactericidal activity

**FIGURE 4:**
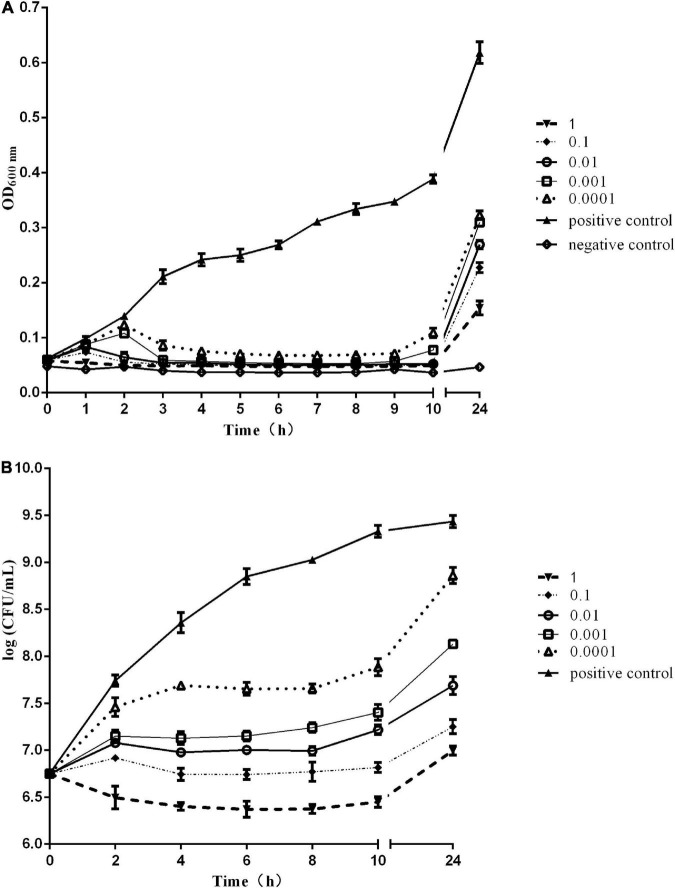
*In vitro* bactericidal activity of phage vB_SabS_Sds2. **(A)** *In vitro* bactericidal activity of phage vB-SabS-Sds2 against *S. abortus equi* S2 strain under five different MOIs (1, 0.1, 0.01, 0.001, and 0.0001); **(B)** Detection of CFUs of phage vB-SabS-Sds2 against *S. abortus equi* S2 under five different MOIs (1, 0.1, 0.01, 0.001, and 0.0001). The data are expressed as means ± SD (*n* = 3).

The *in vitro* bactericidal activity of phage Sds2 against *S. Abortus equi* S2 is shown in Figures 4A,B. The OD_600_ values of the positive control increased continuously within 24 h, and the OD_600_ values of the negative control remained unchanged. At MOI of 1, the OD_600_ values of phage Sds2 decreased rapidly, indicating that bacteria hardly multiplied within 1 hpi (hours post-inoculation). At MOIs of 0.1 and 0.01, the OD_600_ values started to decrease rapidly at 1 hpi. At MOIs of 0.001 and 0.0001, however, the OD_600_ values started to decrease at 2 hpi. During 4–9 hpi, the OD_600_ values of phage Sds2 at all MOIs remained unchanged (Figure 4A). During 4–8 hpi, the numbers of *S. Abortus equi* S2 at different MOIs were correspondingly in an equilibrium of about 10^7^CFU/ml (Figure 4B). At 24 hpi, the OD_600_ values of phage Sds2 at all MOIs increased, but they were still significantly lower than that of the positive control. At MOI of 1 and 0.1, the numbers of *S. Abortus equi* S2 were lower than 10^8^ CFU/ml at 24 hpi. At other MOIs, however, the numbers of *S. Abortus equi* S2 reached 10^9^CFU/ml at 24 hpi. These results indicated that phage Sds2 could remarkably inhibit the growth of *S. Abortus equi* S2 during 2–24 hpi, and its bactericidal activity increased with the increasing MOIs.

### Effect on the abortion rate of phage Sds2 against *Salmonella enterica subspecies enterica serovar abortus equi* challenged mice

During the mice challenge and treatment experiment, clinical symptoms of the mice were observed every 2 h. As shown in Table 1, mice in group F (phage treated) and group G (blank control) showed no abnormal behavior until they gave birth, which indicated that phage Sds2 had no side effects on pregnant mice. Whether the pregnant mice were treated with phage Sds2 before or after the challenge, and given a higher or lower dose, the abortion rates of phage pretreated groups (A and B) and treated groups (C and D) reduced 10∼40% compared to the challenged control mice in group E. Although phage Sds2 did not give 100% inhibition to the challenged mice under the using doses, the higher dose (3.2 × 10^8^ PFU/mouse) showed better effect than the lower dose (3.2 × 10^6^ PFU/mouse). Additionally, the pretreated groups displayed a better inhibitory effect than the treated groups.

**TABLE 1:** Abortion rates of phage Sds2 on pregnant mice challenged with *S. Abortus equi* S2.

Groups^a^	Dose (0.2 ml)		Abortion no. of gestational day (GD)							Abortion rates
	Phage Sds2	*S. Abortus equi* S2	15	16	17	18	19	20	21	
A (phage pretreated)	3.2 × 10^8^ PFU/mouse	2.6 × 10^5^ CFU/mouse					1			1/10
B (phage pretreated)	3.2 × 10^6^ PFU/mouse	2.6 × 10^5^ CFU/mouse						2		2/10
C (phage treated)	3.2 × 10^8^ PFU/mouse	2.6 × 10^5^ CFU/mouse			1		1	1		3/10
D (phage treated)	3.2 × 10^6^ PFU/mouse	2.6 × 10^5^ CFU/mouse		1		1	1		1^b^	4/10
E (challenge control)		2.6 × 10^5^ CFU/mouse			2	2		1	1^b^	6/10
F (phage treated)	5.6 × 10^8^ PFU/mouse									0/10
G (blank control)	0.2 ml LB/mouse									0/10

^*a*^Mice in groups A and B were pretreated with phage Sds2 (3.2 × 10^8^ PFU/mouse, 3.2 × 10^6^ PFU/mouse, respectively) at 1 h before *S. Abortus* equi S2 challenge (2.6 × 10^5^CFU/mouse). Mice in groups C and D were treated with phage Sds2 (3.2 × 10^8^ PFU/mouse, 3.2 × 10^6^ PFU/mouse, respectively) at 1 h after *S. Abortus* equi S2 challenge (2.6 × 10^5^ CFU/mouse). Mice in group E were infected with *S. Abortus* equi S2 (2.6 × 10^5^CFU/mouse). Mice in group F were treated with phage Sds2 at the high dose of 5.6 × 10^8^ PFU/mouse. Mice in group G were given a treatment of LB broth as blank control.

^*b*^The mouse gave birth to a dead fetus.

## Discussion

We identified a novel T5-like phage that can infect *S. Abortus equi*. Although ORFs related to phage replication are clear, some ORFs annotated to be hypothetical proteins may carry accessory genes that enable more efficient phage replication (Fong et al., 2019). Efficient replication may lead to an increased burst size and/or reduced latent period, both of which are expected for phages in biocontrol agents (Pereira et al., 2016). The burst size and latent period of phage Sds2 was 86 PFU/cell and 10 min, respectively, in our study, indicating that phage Sds2 could multiply efficiently.

Phage Sds2 had a wide host range against *Salmonella* and *Escherichia*, which was similar to some T5-like viruses (Lundin et al., 2009). Since the host range of a phage is often limited to one species, phage cocktails are usually used to expand the host spectrum. However, the interference among different phages and high manufacturing costs often limited the production of phage cocktails (Shi et al., 2020). In contrast, polyvalent phages have a stronger ability than phage cocktails in sustaining the diversity of the commensal bacterial community (Niu et al., 2012; Zhao Y. et al., 2019). Phage Sds2 could infect multiple bacterial host species, including *S. Abortus equi*, *Salmonella* serotype, and *E. coli*, and inhibit the growth of *S. Abortus equi* S2 at a wide range of MOIs in the current study. In addition, it is a safe phage that has no lysogeny-related genes, resistance genes, and pathogen-associated genes. Above all, virulent lifestyle, wide host range, and safety indicated that phage Sds2 could be used for biocontrol.

It is difficult to determine phylogenetic relationships through complete genome sequence analysis due to genomic mosaicing in phages (Casjens, 2005). Therefore, the phages are usually classified based on genetic markers. The terminase large subunits are involved in the packaging of the concatemeric DNA in phage capsids, which is one of the most conserved phage proteins, so the terminase large subunits are often used to construct phylogenetic trees and elucidate the evolutionary relationships between different phages (Ahamed et al., 2019; Sui et al., 2021). The major capsid protein is another highly conserved structural protein (Ding et al., 2020). Therefore, the terminase large subunits and the major capsid protein can be used to construct the phylogenetic tree and to reveal evolutionary relationships among different phage families. Although phylogenetic analysis showed that phage Sds2 was most close to coliphage EPS7, while EPS7 was isolated from sewage samples from Seoul and Il-san city, South Korea in the year of 2008, and phage Sds2 was isolated from donkey feces on a donkey farm in Liaocheng city, China in 2019. In addition, they had different host ranges. EPS7 could lyse all the tested 11 *Salmonella typhimurium* strains, while phage Sds2 could lyse all the tested 30 *S. Abortus equi* strains.

Due to phage Sds2 having a wide host spectrum, especially to *S. Abortus equi* strains, it has great potential to control the high prevalence of donkey abortion associated with *S. Abortus equi* infection in China. Before clinical application in donkeys, we first carried out an animal experiment. In the murine model, all the challenged mice did not die or show any obvious symptoms except abortion. A dose of 3.2 × 10^8^ PFU/mouse could provide 90% protection (9/10) to the pregnant mice on 15 GD from abortion. However, these results were inconsistent with the reported research (Wang et al., 2020), and it may be related to the virulence of the challenged bacterial strain, MOI of the tested phage, and immunity of different species of mice (Chen et al., 2019; Wang et al., 2020). These issues inspired us to conduct further studies to address the details in the future.

## Conclusion

A novel virulent bacteriophage vB-SabS-Sds2 with a wide host range was reported. It belonged to the *Tequintavirus*, *Markadamsvirinae*, *Demerecviridae*, *Caudovirales*. Functional annotation of the whole genome indicated that this phage contained no ORFs associated with resistance genes, virulence genes, or lysogeny-related genes. Both genome annotation and phylogenetic analysis indicated that phage Sds2 was highly similar to T5-like bacteriophages. Phage Sds2 exhibited good bactericidal activity against *S. Abortus equi in vitro*. *In vivo* test, Phage Sds2 can reduce the abortive rates of mice challenged with *S. Abortus equi* of both pretreated and treated groups. Our findings highlight the potential of phage Sds2 in preventing the abortion in donkeys caused by *S. Abortus equi*.

## Data availability statement

The datasets presented in this study can be found in online repositories. The names of the repository/repositories and accession number(s) can be found in the article/Supplementary material.

## Ethics statement

The animal study was reviewed and approved by the Animal Welfare and Research Ethics Committee at Qingdao Agricultural University, Shandong, China.

## Author contributions

HR designed the study and analyzed the data together with QP and CZ. LH and PS contributed to the study execution with help from HS and LZ on bacteria collection and reagents supply. JC conducted animal experiments. WL prepared the manuscript. All authors were responsible for data integrity and accuracy of the analysis, contributed to the article, and approved the submitted version.

## Footnotes

1
https://www.most.gov.cn/xxgk/xinxifenlei/fdzdgknr/fgzc/gfxwj/gfxwj2010before/201712/t20171222_137025.html

2
http://rast.nmpdr.org

3
http://opal.biology.gatech.edu/GeneMark/

4
http://www.ncbi.nlm.nih.gov/BLAST

5
http://trna.ucsc.edu/tRNAscan-SE/

## Supplementary material

Supplementary Figure 1 Plagues on the plate of phage vB_SabS_Sds2.

Supplementary Figure 2 Transmission electron microscopy (TEM) image of phage vB_SabS_Sds2.
