# Supplementary figures and images for: Complete genome sequencing of a Tequintavirus bacteriophage with a broad host range against Salmonella Abortus equi isolates from donkeys
Source: Front Microbiol. 2022 Aug 16;13:938616. doi: 10.3389/fmicb.2022.938616 (PMC9424859; doi:10.3389/fmicb.2022.938616)

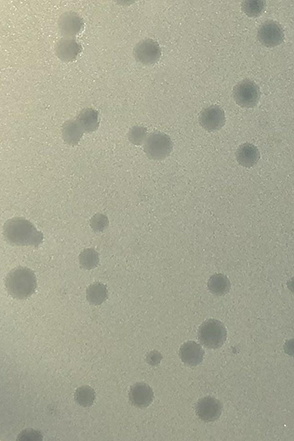

Supplement: Supplementary Figure 1 — Plagues on the plate of phage vB_SabS_Sds2. [file Image_1.TIF]

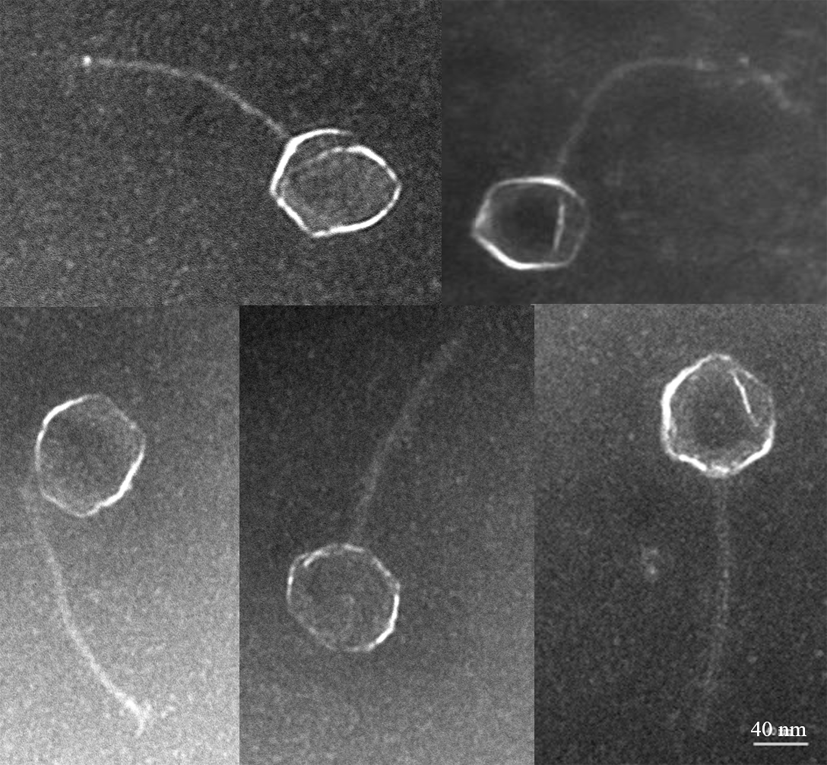

Supplement: Supplementary Figure 2 — Transmission electron microscopy (TEM) image of phage vB_SabS_Sds2. [file Image_2.TIF]

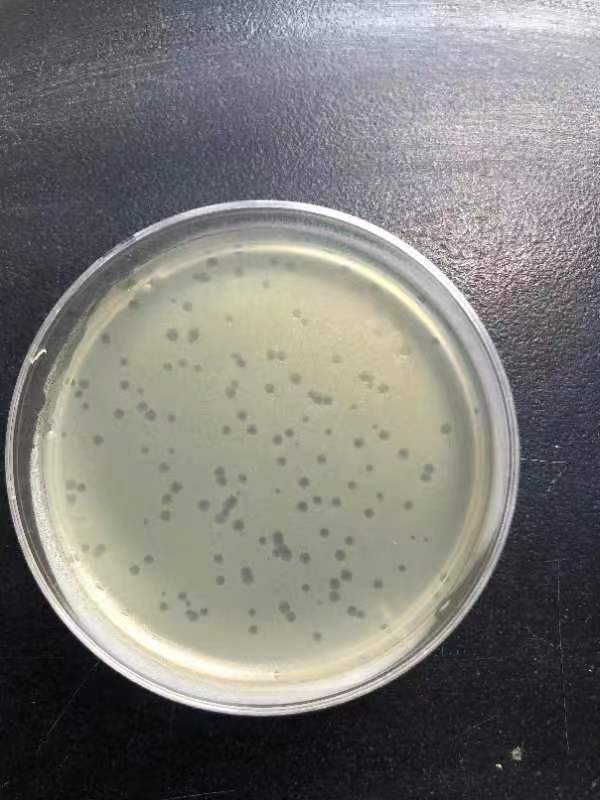

Supplement: Supplementary file 3 [file Image_1.jpg]
