# Supplementary material for: Complete genome sequencing of a Tequintavirus bacteriophage with a broad host range against Salmonella Abortus equi isolates from donkeys
Source: Front Microbiol. 2022 Aug 16;13:938616. doi: 10.3389/fmicb.2022.938616 (PMC9424859; doi:10.3389/fmicb.2022.938616)
Supplement: Supplementary file 5 [file Table_1.docx]

Table S1 Host range of phage vB_SabS_Sds2 to *Salmonella* species and some *E.coli* strains

| No. | Bacteria | Source | | Relative EOP |
| --- | --- | --- | --- | --- |
| 1 | *S. Abortus equi* D1 | | Aborted fetus of donkey, Yanggu, China (2018) | High |
| 2 | *S. Abortus equi* S2 | | Aborted fetus of donkey, Yucheng, China (2018) | High |
| 3 | *S. Abortus equi* D3 | | Aborted fetus of donkey, Donge, China (2018) | High |
| 4 | *S. Abortus equi* D4 | | Aborted fetus of donkey, Neimeng, China (2018) | High |
| 5 | *S. Abortus equi* D5 | | Aborted fetus of donkey, Heilongjiang, China (2018) | High |
| 6 | *S. Abortus equi* D6 | | Aborted fetus of donkey, Xinjiang, China (2018) | Medium |
| 7 | *S. Abortus equi* D7 | | Aborted fetus of donkey, Xizang, China (2019) | High |
| 8 | *S. Abortus equi* D8 | | Aborted fetus of donkey, Jinan, China (2019) | Medium |
| 9 | *S. Abortus equi* D9 | | Aborted fetus of donkey, Yanggu, China (2019) | High |
| 10 | *S. Abortus equi* D10 | | Aborted fetus of donkey, Yucheng, China (2019) | High |
| 11 | *S. Abortus equi* D11 | | Aborted fetus of donkey, Haiyang, China (2019) | High |
| 12 | *S. Abortus equi* D12 | | Aborted fetus of donkey, Donge, China (2019) | Medium |
| 13 | *S. Abortus equi* D13 | | Aborted fetus of donkey, Xinjiang, China (2019) | High |
| 14 | *S. Abortus equi* D14 | | Aborted fetus of donkey, Qinghai, China (2019) | High |
| 15 | *S. Abortus equi* D15 | | Aborted fetus of donkey, Yucheng, China (2020) | Medium |
| 16 | *S. Abortus equi* D16 | | Aborted fetus of donkey, Yuncheng, China (2020) | High |
| 17 | *S. Abortus equi* D17 | | Aborted fetus of donkey, Donge, China (2020) | High |
| 18 | *S. Abortus equi* D18 | | Aborted fetus of donkey, Qihe, China (2020) | High |
| 19 | *S. Abortus equi* D19 | | Aborted fetus of donkey, Guangrao, China (2020) | High |
| 20 | *S. Abortus equi* D20 | | Aborted fetus of donkey, Juncheng, China (2020) | High |
| 21 | *S. Abortus equi* D21 | | Aborted fetus of donkey, Qihe, China (2021) | High |
| 22 | *S. Abortus equi* D22 | | Aborted fetus of donkey, Gucheng, China (2021) | Medium |
| 23 | *S. Abortus equi* D23 | | Aborted fetus of donkey, Jining, China (2021) | High |
| 24 | *S. Abortus equi* D24 | | Aborted fetus of donkey, Donge, China (2021) | High |
| 25 | *S. Abortus equi* D25 | | Aborted fetus of donkey, Liangshan, China (2021) | High |
| 26 | *S. Abortus equi* D26 | | Aborted fetus of donkey, Longnan, China (2021) | Medium |
| 27 | *S. Abortus equi* D27 | | Aborted fetus of donkey, Heze, China (2022) | High |
| 28 | *S. Abortus equi* D28 | | Aborted fetus of donkey, Donge, China (2022) | Medium |
| 29 | *S. Abortus equi* D29 | | Aborted fetus of donkey, Yanggu, China (2022) | Medium |
| 30 | *S. Abortus equi* D30 | | Aborted fetus of donkey, Yucheng, China (2022) | High |
| 31 | S. *sp* F18006 | | Donkey feces, Yucheng, China (2019) | - |
| 32 | S. *sp* F17068 | | Donkey feces, Yanggu, China (2019) | - |
| 33 | S. *sp* F18106 | | Donkey feces, Haiyang, China (2019) | - |
| 34 | S. *sp* F18038 | | Donkey feces, Donge, China (2019) | - |
| 35 | *S. sp* F17014 | | Donkey feces, Yucheng, China (2020) | High |
| 36 | *S. sp* F18066 | | Donkey feces, Juncheng, China (2020) | - |
| 37 | *S. sp* F17060 | | Donkey feces, Donge, China (2020) | - |
| 38 | *S. sp* F59102 | | Donkey feces, Guangrao, China (2020) | - |
| 39 | *S. sp* F18028 | | Donkey feces, Qihe, China (2020) | - |
| 40 | S. *sp* F18024 | | Donkey feces, Jining, China (2021) | - |
| 41 | *S. sp* F18074 | | Donkey feces, Gucheng, China (2021) | High |
| 42 | *S. sp* F18128 | | Donkey feces, Qihe, China (2021) | High |
| 43 | *S. sp* F17188 | | Donkey feces, Jinan, China (2021) | - |
| 44 | *S. sp* F51 | | Donkey feces, Yucheng, China (2021) | - |
| 45 | *S. sp* F467 | | Donkey feces, Donge, China (2021) | - |
| 46 | *S. sp* F171 | | Donkey feces, Longnan, China (2021) | - |
| 47 | *S. sp* F133 | | Donkey feces, Donge, China (2022) | - |
| 48 | *S. sp* F1607 | | Donkey feces, Yanggu, China (2022) | - |
| 49 | *S. sp* F17016 | | Donkey feces, Yucheng, China (2022) | - |
| 50 | *S. Typhi* S1 | | Chicken, Laiyang, China (2018) | High |
| 52 | *S. enterica* S1 | | Chicken, Longkou, China (2018) | - |
| 53 | *S. enterica* C399 | | Chicken, Fushan, China (2018) | - |
| 54 | *S. enterica* C400 | | Chicken, Mihe, China (2018) | High |
| 55 | *S. enterica* C401 | | Chicken, Rushan，China (2018) | - |
| 56 | *S. enterica* C402 | | Chicken, Laixi，China (2018) | - |
| 57 | *S. enterica* C403 | | Chicken, Shouguang, China (2018) | - |
| 58 | *S. enterica* C404 | | Chicken, Laiyang, China (2019) | - |
| 59 | *S. pullorum* C405 | | Chicken, Longkou, China (2019) | Medium |
| 60 | *S. pullorum* C410 | | Chicken, Fushan, China (2019) | Low |
| 61 | *S. pullorum* C411 | | Chicken, Mihe, China (2019) | - |
| 62 | S. *sp* C412 | | Chicken, Rushan, China (2019) | - |
| 63 | S. *sp* C413 | | Chicken, Laixi，China (2019) | - |
| 64 | S. *sp* C414 | | Chicken, Shouguang, China (2019) | - |
| 65 | *S. enterica* C415 | | Chicken, Ningjin, China (2019) | High - |
| 66 | S. *sp* C416 | | Chicken, Changyi, China (2019) | - |
| 67 | S. *sp* C417 | | Chicken, Qihe, China (2019) | - |
| 68 | S. *sp* C418 | | Chicken, Changle, China (2019) | - |
| 69 | S. *sp* C419 | | Chicken, Tanfang, China (2019) | - |
| 70 | S. *sp* C420 | | Chicken, Laiyang, China (2020) | - |
| 71 | *S. pullorum* C421 | | Chicken, Longkou, China (2020) | Low- |
| 72 | S. *sp* C422 | | Chicken, Fushan, China (2020) | - |
| 73 | S. *sp* C423 | | Chicken, Mihe, China (2020) | - |
| 74 | S. *sp* C424 | | Chicken, Rushan, China (2020) | - |
| 75 | *S. enterica* C425 | | Chicken, Laixi，China (2020) | High |
| 76 | S. *sp* C426 | | Chicken, Shouguang, China (2020) | - |
| 77 | S. *sp* C427 | | Chicken, Ningjin, China (2020) | High |
| 78 | S. *sp* C428 | | Chicken, Changyi, China (2020) | - |
| 79 | S. *sp* C436 | | Chicken, Qihe, China (2020) | Medium |
| 80 | S. *sp* C437 | | Chicken, Changle, China (2020) | Medium |
| 81 | S. *sp* C438 | | Chicken, Tanfang, China (2020) | - |
| 82 | S. *sp* C439 | | Chicken, Zibo, China (2020) | High |
| 83 | S. *sp* C440 | | Chicken, Qixia, China (2020) | - |
| 84 | S. *sp* C441 | | Chicken, Penglai, China (2020) | - |
| 85 | *S. enterica* C445 | | Chicken, Liangshan, China (2020) | Low |
| 86 | S. *sp* C446 | | Chicken, Laiyang, China (2021) | - |
| 87 | *S. pullorum* C450 | | Chicken, Fushan, China (2021) | - |
| 88 | S. *sp* C451 | | Chicken, Shanghe, China (2021) | - |
| 89 | *S. pullorum* C452 | | Chicken, Mihe, China (2021) | - |
| 90 | *S. pullorum* C453 | | Chicken, Rushan, China (2021) | - |
| 91 | S. *sp* C454 | | Chicken, Laixi，China (2021) | - |
| 92 | *S. enterica* C455 | | Chicken, Shouguang, China (2021) | High |
| 93 | *S. enterica* C456 | | Chicken, Ningjin, China (2021) | - |
| 94 | S. *sp* p01 | | Pigeon, Chengyang, China (2019) | - |
| 95 | S. *sp* p02 | | Pigeon, Jimo, China (2019) | - |
| 96 | S. *sp* p03 | | Pigeon, Chengyang, China (2020) | - |
| 97 | S. *sp* p04 | | Pigeon, Tanfang, China (2020) | High |
| 98 | S. *sp* p05 | | Pigeon, Zibo, China (2020) | - |
| 99 | S. *sp* p06 | | Pigeon, Qixia, China (2021 | - |
| 100 | S. *sp* p07 | | Pigeon, Penglai, China (2021) | - |
| 101 | S. *sp* p08 | | Pigeon, Qingzhou, China (2021) | Low |
| 102 | S. *sp* s01 | | Swine, Laiyang, China (2018) | - |
| 103 | S. *sp* s02 | | Swine, Fushan, China (2018) | - |
| 104 | S. *sp* s03 | | Swine, Shanghe, China (2018) | - |
| 105 | S. *sp* s04 | | Swine, Mihe, China (2018) | - |
| 106 | S. *sp* s05 | | Swine, Rushan, China (2018) | - |
| 107 | S. *sp* s06 | | Swine, Laixi，China (2018) | - |
| 108 | S. *sp* s07 | | Swine, Laiyang, China (2019) | High |
| 109 | S. *sp* s08 | | Swine, Fushan, China (2019) | Low |
| 110 | S. *sp* s09 | | Swine, Shanghe, China (2019) | - |
| 111 | S. *sp* s10 | | Swine, Mihe, China (2019) | - |
| 112 | S. *sp* s11 | | Swine, Rushan, China (2019) | - |
| 113 | S. *sp* s12 | | Swine, Laixi，China (2019) | - |
| 114 | S. *sp* s13 | | Swine, Laiyang, China (2020) | - |
| 115 | S. *sp* s14 | | Swine, Fushan, China (2020) | - |
| 116 | S. *sp* s15 | | Swine, Shanghe, China (2020) | High |
| 117 | S. *sp* s16 | | Swine, Mihe, China (2020) | - |
| 118 | S. *sp* s17 | | Swine, Rushan, China (2020) | - |
| 119 | S. *sp* s18 | | Swine, Laixi，China (2020) | - |
| 120 | S. *sp* s19 | | Swine, Zibo, China (2020) | - |
| 121 | S. *sp* s20 | | Swine, Qixia, China (2020) | - |
| 122 | S. *sp* s21 | | Swine, Penglai, China (2020) | - |
| 123 | S. *sp* s22 | | Swine, Liangshan, China (2020) | - |
| 124 | S. *sp* s23 | | Swine, Changyi, China (2020) | - |
| 115 | S. *sp* s32 | | Swine, Laiyang, China (2021) | High |
| 116 | S. *sp* s33 | | Swine, Fushan, China (2021) | - |
| 117 | S. *sp* s34 | | Swine, Shanghe, China (2021) | - |
| 118 | S. *sp* s35 | | Swine, Mihe, China (2021) | Low |
| 119 | S. *sp* s36 | | Swine, Rushan, China (2021) | - |
| 120 | S. *sp* s37 | | Swine, Laixi，China (2021) | - |
| 121 | S. *sp* s38 | | Swine, Zibo, China (2021) | High |
| 122 | S. *sp* s39 | | Swine, Qixia, China (2021) | - |
| 123 | S. *sp* s40 | | Swine, Penglai, China (2021) | - |
| 124 | S. *sp* s41 | | Swine, Liangshan, China (2021) | - |
| 125 | *S. pullorum* CVCC533 | | Standard strain | - |
| 126 | *E. coli* D/ND18080281 | | Swine, Fushan, China (2018) | - |
| 127 | *E. coli* D/ND18080285 | | Swine, Laixi，China (2018) | - |
| 128 | *E. coli* ZD/HN190412151 | | Swine, Jimo，China (2018) | High |
| 129 | *E. coli* D/JM18062646 | | Swine, Changyi, China (2019) | Medium |
| 130 | *E. coli* ZD203 | | Swine, Laixi, China (2019) | - |
| 131 | *E. coli* ZD204 | | Swine, Laiyang, China (2020) | - |
| 132 | *E. coli* ZD205 | | Swine, Rushan, China (2020) | - |
| 133 | *E. coli* ZD206 | | Swine, Fushan, China (2021) | - |
| 134 | *E. coli* ZD207 | | Swine, Mihe, China (2021) | - |
| 135 | *E. coli* FD02 | | Mink, Rushan, China (2018) | Medium |
| 136 | *E. coli* FD15 | | Mink, Rongcheng, China (2018) | - |
| 137 | *E. coli* FD28 | | Mink, Chengyang, China (2018) | - |
| 138 | *E. coli* FD35 | | Mink, Zhucheng, China (2018) | - |
| 139 | *E. coli* FD43 | | Mink, Hongdao, China (2018) | - |
| 140 | *E. coli* FD48 | | Mink, Rushan, China (2019) | High |
| 141 | *E. coli* FD53 | | Mink, Chengyang, China (2019) | - |
| 142 | *E. coli* FD58 | | Mink, Penglai, China (2019) | - |
| 143 | *E. coli* FD63 | | Mink, Dalian, China (2019) | - |
| 144 | *E. coli* FD68 | | Mink, Hebei, China (2020) | - |
| 145 | *E. coli* FD73 | | Mink, Xinxiang, China (2020) | Low |
| 146 | *E. coli* FD78 | | Mink, Lianyungang, China (2020) | - |
| 147 | *E. coli* FD83 | | Mink, Rizhao, China (2020) | - |
| 148 | *E. coli* FD88 | | Mink, Juxian, China (2021) | - |
| 149 | *E. coli* FD93 | | Mink, Haiyang, China (2021) | - |
| 150 | *E. coli* FD98 | | Mink, Jinan, China (2021) | - |
| 151 | *E. coli* FD50 | | Mink, Zhucheng, China (2022) | High |
| 152 | K88ac | | Standard strain | Medium |
| 153 | K99 | | Standard strain | Medium |
| 154 | ATCC 25922 | | Standard strain | - |

Notes: “High” stands for EOP ≥ 0.5, “Medium” stands for 0.1≤ EOP <0.5, “Low” stands for 0.001< EOP <0.1, and “-” means EOP ≤ 0.001.
