# Supplementary material for: Complete genome sequencing of a Tequintavirus bacteriophage with a broad host range against Salmonella Abortus equi isolates from donkeys
Source: Front Microbiol. 2022 Aug 16;13:938616. doi: 10.3389/fmicb.2022.938616 (PMC9424859; doi:10.3389/fmicb.2022.938616)
Supplement: Supplementary file 6 [file Table_2.docx]

table S2 Summary of phage Sds2 ORFs with predicted, assigned function

| ORFs | Strand | start | stop | Length | Product | Best-match BLASTp Result | Identities % | E-values | Accession |
| --- | --- | --- | --- | --- | --- | --- | --- | --- | --- |
| ORF1 | - | 70 | 546 | 477 | ribonuclease H | Escherichia phage EPS7 | 100.00 | 1.00E-70 | [YP_001837024.1](https://www.ncbi.nlm.nih.gov/protein/YP_001837024.1?report=genbank&log$=prottop&blast_rank=1&RID=X1B6SDHV014) |
| ORF2 | - | 546 | 815 | 270 | hypothetical protein | Escherichia phage EPS7 | 100.00 | 2e-56 | [YP_001837025.1](https://www.ncbi.nlm.nih.gov/protein/YP_001837025.1?report=genbank&log$=prottop&blast_rank=1&RID=9E6P86HJ013) |
| ORF3 | - | 818 | 1396 | 579 | hypothetical protein | [Escherichia phage EPS7](https://www.ncbi.nlm.nih.gov/Taxonomy/Browser/wwwtax.cgi?id=2886918)  vB_SenS_SB13 | 100.00 | 1e-138 | YP_001837026.1  [YP_001837026.1](https://www.ncbi.nlm.nih.gov/protein/YP_001837026.1?report=genbank&log$=prottop&blast_rank=1&RID=9E6PGGNN013) |
| ORF4 | - | 1495 | 1803 | 309 | hypothetical protein | Salmonella phage 100268_sal2 | 100.00 | 2e-56 | [YP_009320829.1](https://www.ncbi.nlm.nih.gov/protein/YP_009320829.1?report=genbank&log$=prottop&blast_rank=1&RID=WTG7DXEG015) |
| ORF5 | - | 1852 | 2706 | 855 | thymidylate synthase | [Salmonella phage 100268_sal2](https://www.ncbi.nlm.nih.gov/Taxonomy/Browser/wwwtax.cgi?id=1813783) | 99.65 | 0 | [YP_009320830.1](https://www.ncbi.nlm.nih.gov/protein/YP_009320830.1?report=genbank&log$=prottop&blast_rank=2&RID=9EDWNWKM01R) |
| ORF6 | - | 2703 | 3233 | 531 | Dihydrofolate reductase | Salmonella phage 3-29 | 100.00 | 5e-116 | [YP_009818966.1](https://www.ncbi.nlm.nih.gov/protein/YP_009818966.1?report=genbank&log$=prottop&blast_rank=1&RID=9EC00CFA01R) |
| ORF7 | - | 3233 | 4378 | 1146 | Ribonucleotide reductase of class Ia (aerobic), beta subunit | Salmonella phage vB_SenS_SB13 | 99.48 | 0 | [YP_009848075.1](https://www.ncbi.nlm.nih.gov/protein/YP_009848075.1?report=genbank&log$=prottop&blast_rank=2&RID=9EERZ9M2016) |
| ORF8 | - | 4375 | 4872 | 498 | HNH homing endonuclease | [Salmonella phage falkor](https://www.ncbi.nlm.nih.gov/Taxonomy/Browser/wwwtax.cgi?id=2713298) | 99.39 | 8e-120 | [QIO01893.1](https://www.ncbi.nlm.nih.gov/protein/QIO01893.1?report=genbank&log$=prottop&blast_rank=2&RID=9EESCW52013) |
| ORF9 | - | 4954 | 7389 | 2436 | aerobic ribonucleoside diphosphate reductase, large subunit | Salmonella phage bastian | 98.35 | 0.0 | [YP_009858619.1](https://www.ncbi.nlm.nih.gov/protein/YP_009858619.1?report=genbank&log$=prottop&blast_rank=2&RID=9EESJTW5016) |
| ORF10 | - | 7406 | 7648 | 243 | hypothetical protein | [Salmonella phage S113](https://www.ncbi.nlm.nih.gov/Taxonomy/Browser/wwwtax.cgi?id=2231342) | 98.75 | 1e-50 | [YP_009804845.1](https://www.ncbi.nlm.nih.gov/protein/YP_009804845.1?report=genbank&log$=prottop&blast_rank=2&RID=9EEST7P5016) |
| ORF11 | - | 7650 | 8402 | 753 | phosphate starvation inducible protein | Salmonella enterica | 99.20 | 0.0 | [EHV4370628.1](https://www.ncbi.nlm.nih.gov/protein/EHV4370628.1?report=genbank&log$=prottop&blast_rank=2&RID=9EDYXUHG01R) |
| ORF12 | + | 8755 | 10629 | 1875 | anaerobic ribonucleoside-triphosphate reductase | Escherichia phage saus132 | 100.00 | 0.0 | [YP_009794932.1](https://www.ncbi.nlm.nih.gov/protein/YP_009794932.1?report=genbank&log$=prottop&blast_rank=1&RID=9ECTPMM101R) |
| ORF13 | + | 10728 | 11009 | 282 | hypothetical protein | [Escherichia phage EPS7](https://www.ncbi.nlm.nih.gov/Taxonomy/Browser/wwwtax.cgi?id=2886918) | 100.00 | 2e-62 | [YP_001837036.1](https://www.ncbi.nlm.nih.gov/protein/YP_001837036.1?report=genbank&log$=prottop&blast_rank=1&RID=WTY7KHK201N) |
| ORF14 | + | 11019 | 11222 | 204 | hypothetical protein | [Salmonella phage beppo](https://www.ncbi.nlm.nih.gov/Taxonomy/Browser/wwwtax.cgi?id=2713280) | 100.00 | 8e-39 | [QIO00179.1](https://www.ncbi.nlm.nih.gov/protein/QIO00179.1?report=genbank&log$=prottop&blast_rank=1&RID=9ECU6WMB013) |
| ORF15 | + | 11429 | 12205 | 777 | NAD-dependent protein deacetylase of SIR2 family | [Salmonella phage STWB21](https://www.ncbi.nlm.nih.gov/Taxonomy/Browser/wwwtax.cgi?id=2797309) | 100.00 | 0.0 | [QTJ63306.1](https://www.ncbi.nlm.nih.gov/protein/QTJ63306.1?report=genbank&log$=prottop&blast_rank=2&RID=9EDZSDUJ01R) |
| ORF16 | + | 12346 | 12531 | 186 | hypothetical protein | Escherichia phage  VB_Ecos_AKFV33 | 100.00 | 8e-34 | [YP_006382406.1](https://www.ncbi.nlm.nih.gov/protein/YP_006382406.1?report=genbank&log$=prottop&blast_rank=1&RID=WTYKHPD6014) |
| ORF17 | + | 12518 | 13024 | 507 | hypothetical protein | [Salmonella phage LVR16A](https://www.ncbi.nlm.nih.gov/Taxonomy/Browser/wwwtax.cgi?id=2041204) | 100.00 | 5e-110 | [YP_009804394.1](https://www.ncbi.nlm.nih.gov/protein/YP_009804394.1?report=genbank&log$=prottop&blast_rank=1&RID=9ECUSME001R) |
| ORF18 | + | 13027 | 13455 | 429 | hypothetical protein | [Salmonella phage S113](https://www.ncbi.nlm.nih.gov/Taxonomy/Browser/wwwtax.cgi?id=2231342) | 100.00 | 2e-99 | [YP_009804837.1](https://www.ncbi.nlm.nih.gov/protein/YP_009804837.1?report=genbank&log$=prottop&blast_rank=1&RID=9ECUZB9D01R) |
| ORF19 | + | 13465 | 13860 | 396 | hypothetical protein | [Escherichia phage EPS7](https://www.ncbi.nlm.nih.gov/Taxonomy/Browser/wwwtax.cgi?id=2886918) | 100.00 | 3e-75 | [YP_009812260.1](https://www.ncbi.nlm.nih.gov/protein/YP_009812260.1?report=genbank&log$=prottop&blast_rank=1&RID=9EJ85JP6013) |
| ORF20 | + | 14381 | 17266 | 2886 | replication origin binding protein | [Salmonella phage Sw2](https://www.ncbi.nlm.nih.gov/Taxonomy/Browser/wwwtax.cgi?id=2316014) | 100.00 | 0.0 | [YP_009812260.1](https://www.ncbi.nlm.nih.gov/protein/YP_009812260.1?report=genbank&log$=prottop&blast_rank=1&RID=9EJSXVEF013) |
| ORF21 | + | 17250 | 17483 | 234 | hypothetical protein | [Escherichia phage EPS7](https://www.ncbi.nlm.nih.gov/Taxonomy/Browser/wwwtax.cgi?id=2886918) | 100.00 | 1e-47 | [YP_001837047.1](https://www.ncbi.nlm.nih.gov/protein/YP_001837047.1?report=genbank&log$=prottop&blast_rank=1&RID=WTZ6TRWA014) |
| ORF22 | + | 17552 | 18256 | 705 | D2 protein | [Escherichia phage EPS7](https://www.ncbi.nlm.nih.gov/Taxonomy/Browser/wwwtax.cgi?id=2886918) | 100.00 | 7e-160 | [YP_001837048.1](https://www.ncbi.nlm.nih.gov/protein/YP_001837048.1?report=genbank&log$=prottop&blast_rank=1&RID=WTZH7NY1014) |
| ORF23 | + | 18249 | 18512 | 264 | hypothetical protein | [Salmonella phage 100268_sal2](https://www.ncbi.nlm.nih.gov/Taxonomy/Browser/wwwtax.cgi?id=1813783) | 100.00 | 1e-43 | [YP_009320849.1](https://www.ncbi.nlm.nih.gov/protein/YP_009320849.1?report=genbank&log$=prottop&blast_rank=1&RID=WTZNW43S014) |
| ORF24 | + | 18620 | 19030 | 411 | D3 protein | [Escherichia phage EPS7](https://www.ncbi.nlm.nih.gov/Taxonomy/Browser/wwwtax.cgi?id=2886918) | 100.00 | 1e-66 | [YP_001837050.1](https://www.ncbi.nlm.nih.gov/protein/YP_001837050.1?report=genbank&log$=prottop&blast_rank=1&RID=WTZSZ486015) |
| ORF25 | + | 19067 | 19363 | 297 | hypothetical protein | [Epseptimavirus stitch](https://www.ncbi.nlm.nih.gov/Taxonomy/Browser/wwwtax.cgi?id=1540099) | 100.00 | 2e-51 | [YP_009146061.1](https://www.ncbi.nlm.nih.gov/protein/YP_009146061.1?report=genbank&log$=prottop&blast_rank=1&RID=WU08GX1T01N) |
| ORF26 | + | 19414 | 19722 | 309 | transcriptional regulator | [Epseptimavirus stitch](https://www.ncbi.nlm.nih.gov/Taxonomy/Browser/wwwtax.cgi?id=1540099) | 100.00 | 1e-69 | [YP_009146062.1](https://www.ncbi.nlm.nih.gov/protein/YP_009146062.1?report=genbank&log$=prottop&blast_rank=1&RID=WU0E7N6R014) |
| ORF27 | + | 19808 | 20008 | 201 | hypothetical protein | [Salmonella phage JNwz02](https://www.ncbi.nlm.nih.gov/Taxonomy/Browser/wwwtax.cgi?id=2861003) | 100.00 | 9e-38 | [QYC50550.1](https://www.ncbi.nlm.nih.gov/protein/QYC50550.1?report=genbank&log$=prottop&blast_rank=2&RID=9EJBC67R013) |
| ORF28 | + | 20001 | 20972 | 972 | NAD-dependent DNA ligase, subunit A | [Epseptimavirus stitch](https://www.ncbi.nlm.nih.gov/Taxonomy/Browser/wwwtax.cgi?id=1540099) | 100.00 | 0.0 | [YP_009146064.1](https://www.ncbi.nlm.nih.gov/protein/YP_009146064.1?report=genbank&log$=prottop&blast_rank=1&RID=WU0NN4SK015) |
| ORF29 | + | 21175 | 21954 | 780 | DNA ligase, subunit B | Salmonella phage S113 | 99.23 | 0 | [YP_009804827.1](https://www.ncbi.nlm.nih.gov/protein/YP_009804827.1?report=genbank&log$=prottop&blast_rank=2&RID=9EK0BJNX013) |
| ORF30 | + | 21947 | 22714 | 768 | D5 protein | Salmonella phage faergetype | 100.00 | 2e-171 | [YP_009858105.1](https://www.ncbi.nlm.nih.gov/protein/YP_009858105.1?report=genbank&log$=prottop&blast_rank=1&RID=9EMHN219013) |
| ORF31 | + | 22746 | 24269 | 1524 | hypothetical protein | Salmonella phage S113 | 100.00 | 0.0 | YP_009804825.1 [YP_009804825.1](https://www.ncbi.nlm.nih.gov/protein/YP_009804825.1?report=genbank&log$=prottop&blast_rank=1&RID=9EMJ6Y7W016) |
| ORF32 | + | 24266 | 25156 | 891 | DNA primase | Epseptimavirus stitch | 99.66 | 0 | [YP_009146068.1](https://www.ncbi.nlm.nih.gov/protein/YP_009146068.1?report=genbank&log$=prottop&blast_rank=2&RID=9EMP53T8016) |
| ORF33 | + | 25219 | 27786 | 2568 | DNA polymerase | Escherichia phage vB_Eco_mar003J3 | 100.00 | 0.0 | QRV67740.1 [QRV67740.1](https://www.ncbi.nlm.nih.gov/protein/QRV67740.1?report=genbank&log$=prottop&blast_rank=1&RID=9EMJJSF3016) |
| ORF34 | + | 27779 | 28276 | 498 | hypothetical protein | [Epseptimavirus stitch](https://www.ncbi.nlm.nih.gov/Taxonomy/Browser/wwwtax.cgi?id=1540099) | 100.00 | 2e-116 | YP_009146070.1 [YP_009146070.1](https://www.ncbi.nlm.nih.gov/protein/YP_009146070.1?report=genbank&log$=prottop&blast_rank=1&RID=9EMJRXH4013) |
| ORF35 | + | 28273 | 29625 | 1353 | helicase | Epseptimavirus stitch | 99.78 | 0.0 | YP_009146071.1 [YP_009146071.1](https://www.ncbi.nlm.nih.gov/protein/YP_009146071.1?report=genbank&log$=prottop&blast_rank=2&RID=9EMJXHBR016) |
| ORF36 | + | 29766 | 30128 | 363 | hypothetical protein | Escherichia phage EPS7 | 100.00 | 2e-81 | YP_001837061.1 [YP_001837061.1](https://www.ncbi.nlm.nih.gov/protein/YP_001837061.1?report=genbank&log$=prottop&blast_rank=1&RID=9EMK3XPH016) |
| ORF37 | + | 30121 | 30894 | 774 | hypothetical protein | Escherichia phage vB_EcoS-26175I | 99.61 | 1e-173 | [QDK00072.1](https://www.ncbi.nlm.nih.gov/protein/QDK00072.1?report=genbank&log$=prottop&blast_rank=2&RID=9EMBNK9J016) |
| ORF38 | + | 30934 | 31911 | 978 | Phage-associated recombinase | Salmonella phage 118970_sal2 | 100.00 | 0 | [YP_009323864.1](https://www.ncbi.nlm.nih.gov/protein/YP_009323864.1?report=genbank&log$=prottop&blast_rank=1&RID=9ENAFXVM016) |
| ORF39 | + | 31892 | 33730 | 1839 | putative recombination endonuclease, subunit D13 | Salmonella phage Seafire | 100.00 | 0.0 | QRV67746.1 [QRV67746.1](https://www.ncbi.nlm.nih.gov/protein/QRV67746.1?report=genbank&log$=prottop&blast_rank=1&RID=9ENANTYZ016) |
| ORF40 | + | 33734 | 34216 | 483 | hypothetical protein | Salmonella enterica subsp. enterica serovar Derby | 100.00 | 2e-115 | EDA1231026.1 [EDA1231026.1](https://www.ncbi.nlm.nih.gov/protein/EDA1231026.1?report=genbank&log$=prottop&blast_rank=1&RID=9ENAV98J016) |
| ORF41 | + | 34216 | 35091 | 876 | flap endonuclease | Escherichia phage EPS7 | 100.00 | 0 | [YP_001837066.1](https://www.ncbi.nlm.nih.gov/protein/YP_001837066.1?report=genbank&log$=prottop&blast_rank=1&RID=9ENB12CP016) |
| ORF42 | + | 35088 | 35534 | 447 | deoxyuridine 5'-triphosphate nucleotidohydrolase | Salmonella phage S114 | 100.00 | 1e-105 | YP_009804978.1 [YP_009804978.1](https://www.ncbi.nlm.nih.gov/protein/YP_009804978.1?report=genbank&log$=prottop&blast_rank=1&RID=9ENB7SGS016) |
| ORF43 | + | 35512 | 35769 | 258 | hypothetical protein | Escherichia phage DaisyDussoix | 94.12 | 1e-52 | QXV77228.1 [QXV77228.1](https://www.ncbi.nlm.nih.gov/protein/QXV77228.1?report=genbank&log$=prottop&blast_rank=2&RID=9ENBFUNA016) |
| ORF44 | - | 35807 | 38101 | 2295 | Phage tail fiber protein | Salmonella phage  Sepoy | 98.95 | 0 | [YP_009845354.1](https://www.ncbi.nlm.nih.gov/protein/YP_009845354.1?report=genbank&log$=prottop&blast_rank=2&RID=9ENBPCZD013) |
| ORF45 | - | 38145 | 41360 | 3216 | L-shaped tail fiber | Salmonella phage S132 | 93.37 | 0 | [YP_009805601.1](https://www.ncbi.nlm.nih.gov/protein/YP_009805601.1?report=genbank&log$=prottop&blast_rank=2&RID=9ENBZSE1016) |
| ORF46 | - | 41360 | 41782 | 423 | tail protein | Salmonella phage 100268_sal2 | 100.00 | 5e-96 | [YP_009320873.1](https://www.ncbi.nlm.nih.gov/protein/YP_009320873.1?report=genbank&log$=prottop&blast_rank=1&RID=9ENC3CJ7013) |
| ORF47 | - | 41788 | 43845 | 2058 | tail fibers protein | Salmonella phage SH9 | 99.56 | 0 | YP_009804149.1 [YP_009804149.1](https://www.ncbi.nlm.nih.gov/protein/YP_009804149.1?report=genbank&log$=prottop&blast_rank=2&RID=9EP2R8WP01R) |
| ORF48 | - | 43846 | 46695 | 2850 | tail protein | Salmonella phage Sw2 | 100.00 | 1.00E-84 | [ASZ77794.1](https://www.ncbi.nlm.nih.gov/protein/ASZ77794.1?report=genbank&log$=prottop&blast_rank=1&RID=WU46ZEWZ015) |
| ORF49 | - | 46692 | 47306 | 615 | hypothetical protein | Escherichia phage EPS7 | 100.00 | 2e-145 | YP_001837073.1 [YP_001837073.1](https://www.ncbi.nlm.nih.gov/protein/YP_001837073.1?report=genbank&log$=prottop&blast_rank=1&RID=9EP3BZ15013) |
| ORF50 | - | 47415 | 51095 | 3681 | tail fiber protein | Salmonella phage SE24 | 99.84 | 0 | [YP_009848598.1](https://www.ncbi.nlm.nih.gov/protein/YP_009848598.1?report=genbank&log$=prottop&blast_rank=2&RID=9EP3KZBS016) |
| ORF51 | - | 51177 | 51545 | 369 | tape measure chaperone | [Salmonella phage Sw2](https://www.ncbi.nlm.nih.gov/Taxonomy/Browser/wwwtax.cgi?id=2316014) | 100.00 | 2e-84 | [YP_009812293.1](https://www.ncbi.nlm.nih.gov/protein/YP_009812293.1?report=genbank&log$=prottop&blast_rank=1&RID=9EP3WZ22016) |
| ORF52 | - | 51607 | 52011 | 405 | hypothetical protein | Escherichia phage EPS7 | 100.00 | 6e-94 | YP_001837077.1 YP_001837077.1 |
| ORF53 | - | 52008 | 52907 | 900 | minor tail protein | Epseptimavirus stitch | 100.00 | 0 | YP_009146089.1 |
| ORF54 | - | 52912 | 54321 | 1410 | major tail protein | Salmonella phage S113 | 100.00 | 0.0 | YP_009804800.1 |
| ORF55 | - | 54348 | 54833 | 486 | tail terminator protein | Salmonella phage S113 | 99.3 | 2e-115 | YP_009804799.1 |
| ORF56 | - | 54837 | 55604 | 768 | hypothetical protein | Epseptimavirus stitch | 99.61 | 0.0 | YP_009146092.1 |
| ORF57 | - | 55604 | 56116 | 513 | hypothetical protein | Escherichia phage saus132 | 99.41 | 9e-124 | YP_009794978.1 |
| ORF58 | - | 56176 | 57552 | 1377 | major capsid protein | Salmonella phage 100268_sal2 | 100.00 | 0 | YP_009320885.1 |
| ORF59 | - | 57570 | 58202 | 633 | capsid and scaffold protein | Escherichia phage EPS7 | 100.00 | 5e-154 | YP_001837084.1 |
| ORF60 | - | 58206 | 58688 | 483 | Tail fiber protein | Escherichia phage EPS7 | 100.00 | 3e-111 | YP_001837085.1 |
| ORF61 | - | 58685 | 59902 | 1218 | portal protein | Escherichia phage EPS7 | 100.00 | 0.0 | YP_001837086.1 |
| ORF62 | - | 59902 | 60426 | 525 | hypothetical protein | Epseptimavirus stitch | 100.00 | 3e-91 | YP_009146098.1 YP_009146098.1 |
| ORF63 | - | 60454 | 61770 | 1317 | terminase large subunit | Salmonella phage STG2 | 100.00 | 0.0 | YP_009814974.1 |
| ORF64 | - | 61770 | 62252 | 483 | hypothetical protein | Escherichia phage EPS7 | 100.00 | 2e-87 | YP_001837089.1 |
| ORF65 | - | 62263 | 64044 | 1782 | receptor binding protein | Salmonella phage bux | 99.49 | 0.0 | QXV85422.1 |
| ORF66 | + | 64128 | 64394 | 267 | receptor-blocking protein | Epseptimavirus stitch | 100.00 | 3e-50 | YP_009146102.1 |
| ORF67 | + | 64469 | 64783 | 315 | hypothetical protein | Escherichia coli phage vB_EcoS_Ace | 100.00 | 1e-66 | QNR52287.1 |
| ORF68 | + | 64856 | 64960 | 105 | hypothetical protein | Salmonella phage S116 | 100.00 | 2e-04 | YP_009805275.1 |
| ORF69 | + | 64960 | 65205 | 246 | hypothetical protein | Salmonella phage JNwz02 | 98.77 | 5e-35 | QYC50592.1 |
| ORF70 | + | 65202 | 65417 | 216 | hypothetical protein | Salmonella phage Sw2 | 98.59 | 9e-43 | YP_009812312.1 |
| ORF71 | - | 65576 | 66310 | 735 | deoxynucleoside-5'-monophosphatase | Salmonella phage 100268_sal2 | 100.00 | 0 | YP_009320730.1 |
| ORF72 | - | 66398 | 66790 | 393 | hypothetical protein | Salmonella phage 100268_sal2 | 100.00 | 9e-91 | YP_009320731.1 |
| ORF73 | - | 66823 | 67101 | 279 | hypothetical protein | Escherichia phage EPS7 | 100.00 | 1e-61 | YP_001836926.1 |
| ORF74 | - | 67154 | 68818 | 1665 | putative A1 protein | Salmonella phage faergetype | 99.64 | 0.0 | YP_009858064.1 |
| ORF75 | - | 68928 | 69152 | 225 | hypothetical protein | Salmonella phage Sw2 | 100.00 | 2e-30 | YP_009812141.1 |
| ORF76 | - | 69203 | 69619 | 417 | A2 protein | Salmonella phage S126 | 100.00 | 2e-80 | YP_009805424.1 |
| ORF77 | - | 69719 | 69970 | 252 | hypothetical protein | Escherichia phage EPS7 | 100.00 | 4e-53 | YP_001836929.1 |
| ORF78 | - | 70136 | 70345 | 210 | hypothetical protein | Salmonella phage Seafire | 100.00 | 1e-41 | YP_009816657.1 |
| ORF79 | - | 70342 | 70500 | 159 | hypothetical protein | Salmonella phage Seafire | 100.00 | 2e-30 | YP_009816658.1 |
| ORF80 | + | 71578 | 72579 | 1002 | hypothetical protein | Epseptimavirus stitch | 98.50 | 0.0 | YP_009145950.1 |
| ORF81 | + | 72644 | 73138 | 495 | hypothetical protein | Salmonella phage Sepoy | 100.00 | 7e-102 | YP_009845210.1 |
| ORF82 | + | 73246 | 73476 | 231 | hypothetical protein | Salmonella phage Sepoy | 98.68 | 7e-48 | YP_009845211.1 |
| ORF83 | + | 73530 | 73742 | 213 | hypothetical protein | Salmonella phage JNwz02 | 100.00 | 1e-42 | QYC50605.1 |
| ORF84 | + | 73852 | 74196 | 345 | hypothetical protein | Salmonella phage STG2 | 98.25 | 4e-77 | YP_009815114.1 |
| ORF85 | + | 74193 | 74393 | 201 | hypothetical protein | Escherichia phage EPS7 | 100.00 | 7e-40 | YP_001836937.1 |
| ORF86 | + | 74517 | 74756 | 240 | hypothetical protein | Salmonella phage Shivani | 97.47 | 6e-51 | YP_009194660.1 |
| ORF87 | - | 76299 | 76655 | 357 | hypothetical protein | Salmonella phage S113 | 100.00 | 8e-82 | YP_009804937.1 |
| ORF88 | - | 76642 | 77373 | 732 | hypothetical protein | Salmonella phage S113 | 99.59 | 6e-178 | YP_009804936.1 |
| ORF89 | - | 77373 | 77987 | 615 | hypothetical protein | Salmonella phage 100268_sal2 | 99.02 | 6e-149 | YP_009320745.1 |
| ORF90 | - | 77987 | 78172 | 186 | hypothetical protein | Epseptimavirus stitch | 100.00 | Be-17 | YP_009145959.1 |
| ORF91 | - | 78382 | 78924 | 543 | hypothetical protein | Salmonella phage vB STyj5-1 | 100.00 | 2e-122 | QQV89276.1 |
| ORF92 | - | 78945 | 79196 | 252 | capsid and scaffold protein | Salmonella phage OSY-STA | 100.00 | 1e-50 | YP_009851855.1 |
| ORF93 | - | 79270 | 79737 | 468 | hypothetical protein | Epseptimavirus stitch | 100.00 | 4e-112 | YP_009145963.1 |
| ORF94 | - | 79734 | 79934 | 201 | hypothetical protein | Epseptimavirus stitch | 100.00 | 3e-41 | YP_009145964.1 |
| ORF95 | - | 80035 | 80367 | 333 | hypothetical protein | Escherichia phage saus132 | 100.00 | 5e-74 | YP_009794855.1 |
| ORF96 | - | 80357 | 80602 | 246 | hypothetical protein | Escherichia phage EPS7 | 98.77 | 6e-48 | YP_001836953.1 |
| ORF97 | - | 80599 | 80880 | 282 | hypothetical protein | Salmonella phage S114 | 98.92 | 3e-39 | YP_009805088.1 |
| ORF98 | - | 80880 | 81293 | 414 | hypothetical protein | Salmonella phage phagemcphageface | 98.54 | 4e-94 | QIN99227.1 |
| ORF99 | - | 81293 | 81544 | 252 | hypothetical protein | Escherichia phage JLBYU43 | 100.00 | 7e-52 | UGO55759.1 |
| ORF100 | - | 81623 | 82054 | 432 | hypothetical protein | Epseptimavirus stitch | 100.00 | 1e-100 | YP_009145970.1 |
| ORF101 | - | 82216 | 82806 | 591 | putative serine/threonine protein phosphatase | Salmonella phage LVR16A] | 100.00 | 3e-142 | YP_009804316.1 |
| ORF102 | - | 82806 | 83012 | 207 | hypothetical protein | Salmonella phage SH9] | 100.00 | 2e-41 | YP_009804249.1 |
| ORF103 | - | 83012 | 83380 | 369 | hypothetical protein | Salmonella phage LVR16A] | 100.00 | 8e-83 | YP_009804318.1 |
| ORF104 | - | 83380 | 84243 | 864 | serine/threonine protein phosphatase | Epseptimavirus stitch | 100.00 | 0 | QRV67811.1 |
| ORF105 | - | 84243 | 84623 | 381 | hypothetical protein | Salmonella enterica subsp. enterica | 100.00 | 3e-70 | EDY0344168.1 |
| ORF106 | - | 84726 | 85016 | 291 | thioredoxin | Epseptimavirus stitch | 100.00 | 1e-64 | YP_009145977.1 |
| ORF107 | - | 85009 | 85419 | 411 | hypothetical protein | Epseptimavirus stitch | 100.00 | 5e-83 | YP_009145978.1 |
| ORF108 | - | 85495 | 85911 | 417 | hypothetical protein | Salmonella phage 100268_sal2 | 100.00 | 5e-95 | YP_009320766.1 |
| ORF109 | - | 85987 | 86400 | 414 | endolysin | Salmonella phage 100268_sal2 | 100.00 | 4e-96 | YP_009320767.1 |
| ORF110 | - | 86397 | 87053 | 657 | holin | Epseptimavirus stitch | 100.00 | 5e-159 | YP_009145981.1 |
| ORF111 | - | 87210 | 87809 | 600 | ATP-dependent Clp protease | Escherichia phage EPS7 | 100.00 | 4e-148 | YP_001836968.1 |
| ORF112 | - | 87822 | 88574 | 753 | dNMP kinase | Escherichia virus EPS7 | 100.00 | 0 | YP_001836969.1 |
| ORF113 | - | 88858 | 89307 | 450 | hypothetical protein | Escherichia virus EPS7 | 100.00 | 9e-91 | YP_001836971.1 |
| ORF114 | - | 89264 | 89962 | 699 | hypothetical protein | Escherichia phage EPS7 | 99.57 | 5e-171 | YP_001836972.1 |
| ORF115 | - | 90108 | 90455 | 348 | hypothetical protein | Salmonella phage Sw2 | 99.13 | 2e-77 | YP_009812185.1 |
| ORF116 | - | 90572 | 90856 | 285 | hypothetical protein | Salmonella phage S126 | 98.94 | 1e-48 | YP_009805387.1 |
| ORF117 | - | 91103 | 91522 | 420 | hypothetical protein | Salmonella phage vB_SenS-3 | 97.84 | 3e-86 | QIN93389.1 |
| ORF118 | - | 91515 | 91814 | 300 | hypothetical protein | Salmonella phage S113 | 100.00 | 5e-65 | YP_009804900.1 |
| ORF119 | - | 91814 | 92086 | 273 | hypothetical protein | Salmonella phage 100268_sal2] | 98.89 | 3e-57 | YP_009320779.1 |
| ORF120 | - | 92163 | 92558 | 396 | hypothetical protein | Epseptimavirus stitch] | 100.00 | 2e-72 | YP_009145996.1 |
| ORF121 | - | 92617 | 92802 | 186 | hypothetical protein | Salmonella phage 100268_sal2 | 100.00 | 2e-24 | YP_009320781.1 |
| ORF122 | - | 92867 | 93022 | 156 | hypothetical protein | Escherichia phage EPS7 | 97.44 | 9e-18 | YP_001836982.1 |
| ORF123 | - | 93242 | 93610 | 369 | Ribonucleotide reductase subunit | Epseptimavirus stitch] | 100.00 | 2e-72 | YP_009145996.1 |
| ORF124 | - | 93982 | 94176 | 195 | hypothetical protein | Salmonella phage S116 | 98.44 | 2e-37 | YP_009805220.1 |
| ORF125 | - | 94374 | 94667 | 294 | hypothetical protein | Salmonella phage 100268_sal2 | 100.00 | 9e-64 | YP_009320785.1 |
| ORF126 | - | 94827 | 94991 | 165 | hypothetical protein | Yersinia phage phiR2-01 | 100.00 | 5e-29 | YP_007237029.1 |
| ORF127 | - | 94984 | 95205 | 222 | hypothetical protein | Salmonella phage 100268_sal2 | 100.00% | 3e-46 | YP_009320787.1 |
| ORF128 | - | 95468 | 95668 | 201 | hypothetical protein | Salmonella phage 100268_sal2 | 100.00 | 9e-40 | YP_009320788.1 |
| ORF129 | - | 96326 | 96529 | 204 | hypothetical protein | Salmonella phage GEC_vB_N7 | 100.00 | 8e-42 | QPI15523.1 |
| ORF130 | - | 96717 | 96893 | 177 | hypothetical protein | Salmonella phage SH9 | 98.28 | 2e-33 | YP_009804222.1 |
| ORF131 | - | 97441 | 97794 | 354 | hypothetical protein | Salmonella phage bastian | 100.00 | 2e-79 | YP_009858659.1 |
| ORF132 | - | 98027 | 98344 | 318 | hypothetical protein | Epseptimavirus stitch | 99.05 | 6e-72 | YP_009146007.1 |
| ORF133 | - | 98443 | 98607 | 165 | hypothetical protein | Salmonella phage Seafire | 100.00 | 5e-28 | YP_009816723.1 |
| ORF134 | - | 98795 | 98953 | 159 | hypothetical protein | Salmonella phage Seafire | 100.00 | 5e-28 | YP_009816723.1 |
| ORF135 | - | 100201 | 100386 | 186 | hypothetical protein | Salmonella phage SH9 | 100.00 | 1e-22 | YP_009804217.1 |
| ORF136 | - | 100830 | 101054 | 225 | hypothetical protein | Salmonella phage 100268_sal2 | 93.24 | 1e-42 | YP_009320801.1 |
| ORF137 | - | 101105 | 101377 | 273 | hypothetical protein | Epseptimavirus stitch | 98.89 | 2e-54 | YP_009146012.1 |
| ORF138 | - | 101926 | 102201 | 276 | hypothetical protein | Salmonella enterica | 98.90 | 2e-58 | EHV4370661.1 |
| ORF139 | - | 102292 | 102498 | 207 | hypothetical protein | Salmonella phage vB_SenS-3 | 100.00 | 2e-40 | QIN93431.1 |
| ORF140 | - | 102491 | 102634 | 144 | hypothetical protein | Epseptimavirus stitch | 100.00 | 2e-26 | YP_009146015.1 |
| ORF141 | - | 102735 | 103073 | 339 | hypothetical protein | Salmonella phage SH9 | 100.00 | 5e-60 | YP_009804211.1 |
| ORF142 | - | 103075 | 103260 | 186 | Phage antitermination protein | Epseptimavirus stitch | 100.00 | 6e-37 | YP_009146017.1 |
| ORF143 | - | 103506 | 103802 | 297 | hypothetical protein | Escherichia phage EPS7 | 100.00 | 1e-65 | YP_001837007.1 |
| ORF144 | - | 103909 | 104586 | 678 | nicotinamide mononucleotide transporter | Escherichia phage EscoHU1 | 99.11 | 7e-142 | BDC47531.1 |
| ORF145 | - | 104588 | 105643 | 1056 | Ribosylnicotinamide kinase | Salmonella phage Sepoy | 98.86 | 0 | YP_009146021.1 |
| ORF146 | + | 105909 | 106895 | 987 | hypothetical protein | Epseptimavirusstitch | 99.70 | 3e-175 | YP_009146021.1 |
| ORF147 | + | 106918 | 107334 | 417 | hypothetical protein | Salmonella phage Sepoy] | 100.00 | 9e-38 | YP_009845290.1 |
| ORF148 | - | 107655 | 108599 | 945 | hypothetical protein | Epseptimavirus stitch | 100.00 | 6e-177 | YP_009146023.1 |
| ORF149 | - | 108611 | 108811 | 201 | hypothetical protein | Escherichia phage EPS7 | 100.00 | 4e-38 | YP_001837011.1 |
| ORF150 | - | 109777 | 110226 | 450 | hypothetical protein | Salmonella phage 3-29 | 100.00 | 4e-104 | YP_009818950.1 |
| ORF151 | - | 110226 | 110396 | 171 | hypothetical protein | Escherichia phage EPS7 | 100.00 | 3e-30 | YP_001837013.1 |
| ORF152 | - | 110465 | 110914 | 450 | spore cortex-lytic enzyme precursor | Epseptimavirus stitch | 100.00 | 1e-106 | YP_009146027.1 |
| ORF153 | - | 110920 | 111237 | 318 | hypothetical protein | Salmonella phage 100268_sal2 | 100.00 | 4e-71 | YP_009320818.1 |
| ORF154 | - | 111677 | 112315 | 639 | Phage tail fiber protein | Salmonella phage LVR16A | 100.00 | 2e-150 | YP_009804371.1 |
| ORF155 | - | 112370 | 112552 | 183 | hypothetical protein | Salmonella phage 100268_sal2 | 98.33 | 4.00E-33 | YP_009320820.1 |
| ORF156 | - | 112623 | 113324 | 702 | putative metallopeptidase | Salmonella phage SH9 | 99.57 | 2.00E-174 | YP_009804198.1 |
| ORF157 | - | 113355 | 113567 | 213 | hypothetical protein | Epseptimavirus stitch | 97.14 | 7.00E-42 | YP_009146032.1 |
| ORF158 | - | 113609 | 113824 | 216 | Phage tail length tape-measure protein | Escherichia virus EPS7 | 98.59 | 5.00E-40 | YP_001837021.1 |
| ORF159 | - | 113886 | 114401 | 516 | hypothetical protein | Salmonella phage LVR16A | 100.00 | 3.00E-112 | YP_009804376.1 |
| ORF160 | - | 114485 | 114769 | 285 | hypothetical protein | Epseptimavirus stitch | 98.91 | 6.00E-58 | YP_009804376.1 |
