# Supplementary material for: Complete genome sequencing of a Tequintavirus bacteriophage with a broad host range against Salmonella Abortus equi isolates from donkeys
Source: Front Microbiol. 2022 Aug 16;13:938616. doi: 10.3389/fmicb.2022.938616 (PMC9424859; doi:10.3389/fmicb.2022.938616)
Supplement: Supplementary file 7 [file Table_3.docx]

| NO | tRNAs | strand | start | stop | sequence |
| --- | --- | --- | --- | --- | --- |
| 1 | tRNA-Met-CAT | - | 94290 | 94218 | agttagttggcagagtggttatgcacctccttcatacggagcgactacagtggttcaactccactactaacta |
| 2 | tRNA-Ile-GAT | - | 94372 | 94300 | gctctgatagttcaacaggttagaacaggcgaccgataatcgtcaaatcttggttcgatcccaagtcggagta |
| 3 | tRNA-Gly-TCC | - | 95295 | 95224 | gcgtgattagttcagtggctagaataactggcttccacccagtagacacgagttcgactctcgtatcccgca |
| 4 | tRNA-Gln-TTG | - | 95377 | 95305 | tggagagtagtgtaacggttagcacaacggcctttgactccgttaatggtaggttcgattcctccttctccag |
| 5 | tRNA-Gln-CTG | - | 95459 | 95387 | tgggatgtagatcaattggcagatcgtcggcctctgactccgaaggttccacgttcgatccgtggcatcccag |
| 6 | tRNA-Arg-ACG | - | 95767 | 95696 | gcgtccttatttcaacggaaagaatgtaaagccacgaactttacgatcggggttcgattccctgaggatgca |
| 7 | tRNA-Pseudo-TGA | - | 96637 | 96551 | ggaaggtaggacatagtggtatgtaacaggtcttgaaaacctgcccgctgtagcgatatggtgatggttcgactccattaccttcct |
| 8 | tRNA-Leu-TAG |  | 96990 | 96913 | ggggatgtggcgaaattggcagacgcgctagatttaggttctagtcttcgggtgtgggttcgagtccctccatcccta |
| 9 | tRNA-Ala-TGC | - | 97073 | 97001 | ggggtcataggttatttggttaaacttactgccttgcaagcagtggaactcagttcaattctgagtgactcca |
| 10 | tRNA-Val-TAC | - | 97893 | 97823 | gctcggttagtttaatgggagaaccccgtctttacacggcggttgcgatagttcgattctatcaccgagta |
| 11 | tRNA-Lys-TTT | - | 98440 | 98365 | agatcgctagctcaattggtttagcagcacccggcttttaaccggaaggttctgggttcgagtcccaggcggtcta |
| 12 | tRNA-Met-CAT | - | 98706 | 98632 | tgcgggttagatctctggtagagatcgctagtctcataagctagaaagaggtaggttcgattcctgcacccgctt |
| 13 | tRNA-Pro-TGG | - | 98790 | 98716 | ctccgtgtagctcagtttggccagagcgttccgtttggggcggtagggtcgggggttcaaatcctcccacggaga |
| 14 | tRNA-Gly-GCC | - | 99341 | 99271 | gcgttcgtagttaaaaggtataatttttggttgccaaccagaagttgagggttcgattccctccgaccgca |
| 15 | tRNA-Lys-CTT | - | 99557 | 99483 | gcatctgtagcttagttgacttaaagcaaccgactcttaatcggaagatcctgagtttgaatctcagcaggtgta |
| 16 | tRNA-Asn-GTT | - | 100571 | 100492 | gggtcgttagccaagcggtttggcggcggattgttaatccgtgtcgaaagacaacgtaggttcgaatcctacacggcccg |
| 17 | tRNA-Cys-GCA | - | 100655 | 100583 | cgaccgttggctgaatggcttaggcggaggcctgcaaaacctccttatgtgagttcgaatctcatgcggtcgt |
| 18 | tRNA-Glu-TTC | - | 101637 | 101564 | gtcctgttagacaaactggtaaagtcactaccctttcaaggtaggatttgcgggttcgatccccgcacaggacg |
| 19 | tRNA-Pseudo-GTA | - | 101735 | 101648 | gttggattagtatcgtagaggtagcgaagcagactgtaaatctgccgactcggaagggtctcgggtggttcgactccatcatccaaca |
| 20 | tRNA-Leu-TAA | - | 102726 | 102653 | aggggtgtaatcgaattggcataggtactggacttaaaattcaggttttgtgggttcgaatcccaccaccccta |
| 21 | tRNA-Met-CAT | - | 103355 | 103281 | ggttctcaagctcatttggtatgagccgtcgcctcataagcgaaaggtaggtaggttcgaatcctccgggagcca |
| 22 | tRNA-Arg-TCT | - | 108912 | 108841 | cggggtgtagtctaagggataggcaggagtcttctaaattcctttatgcaggttcgaatcctgtcacctcgg |

Table S3 tRNAs in the genome of phage Sds2
